# Editorial: Sexual violence in times of conflict

**DOI:** 10.3389/fpubh.2025.1637494

**Published:** 2025-06-26

**Authors:** Thomas Wenzel, Jan Ilhan Kizilhan, Thomas G. Schulze

**Affiliations:** ^1^World Psychiatric Association Psychological Aspects of Persecution and Torture, Geneva, Switzerland; ^2^Department of Psychiatric Phänomik and Genomic (IPPG), Ludwig Maximilians University, Munich, Germany; ^3^Department of Psychiatry and Psychotherapy, Medical University of Vienna, Vienna, Austria; ^4^Institute for Transcultural Psychotherapy (ITP), Baden-Wuerttemberg Cooperative State University (DHBW), Stuttgart, Germany; ^5^Dohuk University, Duhok, Iraq; ^6^World Psychiatric Association, Geneva, Switzerland

**Keywords:** gender, posttraumatic stress disorder (PTSD), war, torture, FGM

Sexual violence has been demonstrated to be the form of violence probably most strongly associated with severe and long-term suffering, with potentially life-long symptoms, but also with a broader impact on family, community, and members of the next generation (1, 2). Further consequences include rising suicide rates (3), especially when victims get pregnant from rape and face the difficult situation of stigma and of taking care of the resulting offspring in an often hostile environment. Physical injury and infections (4) can also accompany the fate of survivors and should be considered in addition to the psychological, economic, and social impact.

The importance of the issue is reflected, for example, by the inclusion of sexual violence as a special focus and issue of concern in international human rights and humanitarian law standards^1^.

The term “sexual violence” can be used regarding different situations, ranging from sexual assault, rape and sexual torture to harassment and other forms of sexual violence, all unfortunately common events in apparently “lawless” conflict- and post-conflict settings and common even in early records of that events, or even as a systematic strategy of terror (5–8). “Virginity testing”, forced marriage, domestic violence, or FGM (9) also should be considered in this context, though they most commonly occur in “everyday” settings, but also with increased frequency in conflict environments.

Sexual violence has become a key question in the context of setting limits in armed conflicts, also considering its increasing global frequency. This rise in frequency can be attributed not only to the growing number of such conflicts and deterioration of human rights standards in some countries, but also to its increased use by state and non-state actors such as ISIS. The last was explored, for example, by Kizilhan et al. (10, 11) in regard to the persecuted Kurdish Yezidi minority of Iraq in the context of the ongoing genocidal environment experienced by this group.

The persistent work of the international courts to prosecute the crime of sexual violence is a good additional indicator of the attention given internationally to the criminal aspects of sexual violence perpetrated in conflicts, together with the establishment of specialized expert teams by the UN.

In this context, it should be considered that collection of data in armed conflict or similar situations can be a major challenge, because of the re-traumatisation risk of victims, but also because of the often disorganized or even dangerous research environments. The courage and dedication of researchers, as well as the victims giving testimony in such situations, lend additional importance to the results.

The authors in this special issue, therefore, take up insufficiently covered questions and situations in that important field.

Michael and Demeke report on the recent explosive situation in the Amhara region of Ethiopia using a multicenter-based cross-sectional design.

Green et al. report on the situation of health care workers supporting Rohingya victims of sexual and other violence in the vast refugee camp of Cox‘s Bazaar, one of the largest global camps, drawing attention to that largely neglected crisis point.

Kidie et al. use a mixed methods approach, probably the best strategy to better understand both the scope and the experience of victimization after sexualized violence, again using a group of survivors in the emerging Ethiopian crisis region.

Baroudi et al. demonstrated the prevalence of sexual violence as a problem to be addressed also in those persons living in exile, usually after escaping the original war and persecution environments.

The editors hope that the results from the research presented in this special volume will further underline the need to fight against any form of sexual violence, especially in conflict regions, but also to offer sustainable immediate as well as long-term and interdisciplinary support and protection to all survivors.

## Footnotes

^1^https://documents.un.org/doc/undoc/gen/g16/000/97/pdf/g1600097.pdf (Accessed April 20, 2025).
